# Distribution of Minor and Major Metallic Elements in Residential Indoor Dust: A Case Study in Latvia

**DOI:** 10.3390/ijerph20136207

**Published:** 2023-06-22

**Authors:** Agnese Araja, Maris Bertins, Gunita Celma, Lauma Busa, Arturs Viksna

**Affiliations:** Faculty of Chemistry, University of Latvia, Jelgavas Str.1, LV-1004 Riga, Latvia

**Keywords:** indoor dust, metallic elements, dust sampling, ICP-MS, PCA

## Abstract

The coronavirus disease 2019 (COVID-19) pandemic has not only brought considerable and permanent changes to economies and healthcare systems, but it has also greatly changed the habits of almost the entire society. During the lockdowns, people were forced to stay in their dwellings, which served as a catalyst for the initiation of a survey on the estimation of the metallic element content in residential indoor dust in different parts of Latvia. This article presents the study results obtained through the analysis of collected dust samples from 46 dwellings, both in the capital of Latvia, Riga, and in smaller cities. Two methods were employed for indoor dust collection: vacuum sampling and manual sampling with a brush and plastic spatula. After microwave-assisted acid extraction, the samples were analyzed using inductively coupled plasma mass spectrometry (ICP-MS) in terms of the major (Na, K, Ca, Mg, Al and Fe) and minor (Mn, Ni, Co, Pb, Cr, As, Ba, Li, Be, B, V, Cu, Zn, Se, Rb, Sr, Cd, La, Ce and Bi) elements. For the data analysis, principal component analysis was performed. Among the measured metals, the highest values were determined for the macro and most abundant elements (Na > K > Ca > Fe > Mg > Al). The concentration ranges of the persistently detected elements were as follows: Pb, 0.27–1200 mg kg^−1^; Cd, 0.01–6.37 mg kg^−1^; Ni, 0.07–513 mg kg^−1^; As, 0.01–69.2 mg kg^−1^; Cu, 5.71–1900 mg kg^−1^; Zn, 53.6–21,100 mg kg^−1^; and Cr, 4.93–412 mg kg^−1^. The critical limit values of metallic elements in soil defined by the legislation of the Republic of Latvia (indicating the level at or above which the functional characteristics of soil are disrupted, or pollution poses a direct threat to human health or the environment) were exceeded in the following numbers of dwellings: Pb = 4, Ni = 2, As = 1, Cu = 16, Cr = 1 and Zn = 28.

## 1. Introduction

Exposure to indoor air pollutants has a significant impact, firstly, since it has been reported that people in industrialized countries spend an average of 90% of their time in enclosed microenvironments [1,2]. Secondly, in accordance with the World Health Organization (WHO) [3], indoor microenvironment air contamination is one of the main current environmental health risks, as indoor environments frequently have even higher levels of air pollutants than the outdoor environment [4,5]. Recent studies [6,7,8,9] have demonstrated the extent of worldwide interest in human exposure to indoor pollutants; in particular, residential dust may be considered one of the important pathways of human exposure to toxic trace elements. Turner defined household dust as fine (≤100 µm) settled or airborne particulate material encountered in the indoor domestic setting [10]. However, it must be pointed out that household dust is a heterogeneous mixture of materials of different sizes and shapes, organic and inorganic in origin, including different ultrafine fibers, mold, allergens, soot, animal fur, skin particles, and heating and building residues [11]. Indoor dust is considered a complex matrix in terms of its numerically remarkable possible origin sources, including the infiltration of outdoor contaminants, heating, cooking and household electronic devices, building and reconstruction materials, consumer products, smoking and incense burning, and other sources, which might be closely linked to inhabitants’ activities [12,13,14]. The ability of indoor dust to act as both a sink and a transport medium for various persistent chemical contaminants, such as heavy metals, has been demonstrated in many studies [15,16,17]. Foremost among the various other contaminants detected in residential dust, heavy metals are of well-grounded concern due to long-term exposure, their adverse health effects and such specific features as their high toxicity, non-biodegradability, persistence, bioaccumulation potential and long biological half-life [18,19]. It has been suggested that household dust-bound heavy metals accumulate in humans through inhalation, non-dietary ingestion or dermal contact. The absorption and penetration of metals into human fatty tissues and circulatory system tissues, as well as other parts of the body, will probably result in metal-associated diseases later in life [20,21,22]. Children at a young age are more frequently exposed to interior contaminants than people in other age groups due to their particular behaviors, such as crawling and hand-to-mouth practices. The intensive growth and rapid development of children’s organs in the first years of life and their immature immune system, higher inhalation rates per body mass and lower tolerance to pollutants make children much more susceptible to heavy metal exposure than adults [23,24].

This is the first study to describe the analysis results of 120 indoor dust samples gathered from 46 residential dwellings in Latvia in terms of their metallic element concentrations. In this paper, we report the concentrations of 25 metallic elements in milligrams per kilogram (mg kg^−1^) of total dust in the particle size range < 0.2 mm, emphasizing the concentrations of non-carcinogenic elements and carcinogenic elements such as copper (Cu), nickel (Ni), zinc (Zn), manganese (Mn), arsenic (As), cadmium (Cd), chromium (Cr) and lead (Pb), as these elements present greater health risks for both adults and children [23]. The objective of this study was to determine the content of 25 major, or most abundant, and minor, or trace, metallic elements in residential indoor dust in Latvia. Overall, this study targets to answer the following questions:

- Which are the minor and major metallic elements in residential indoor dust in Latvia?
- Which are the concentration ranges of toxic elements adhered to the investigated household dust from dwellings located in urban, suburban and rural areas in Latvia?
- Can the results from two non-standardized dust-sampling approaches—dust vacuuming and manual dust sampling with a brush and plastic spatula—be comparable?
- Which trace elements detected in residential indoor dust could be of greater concern for human health?

## 2. Materials and Methods

### 2.1. Study Area

**Figure 1:**
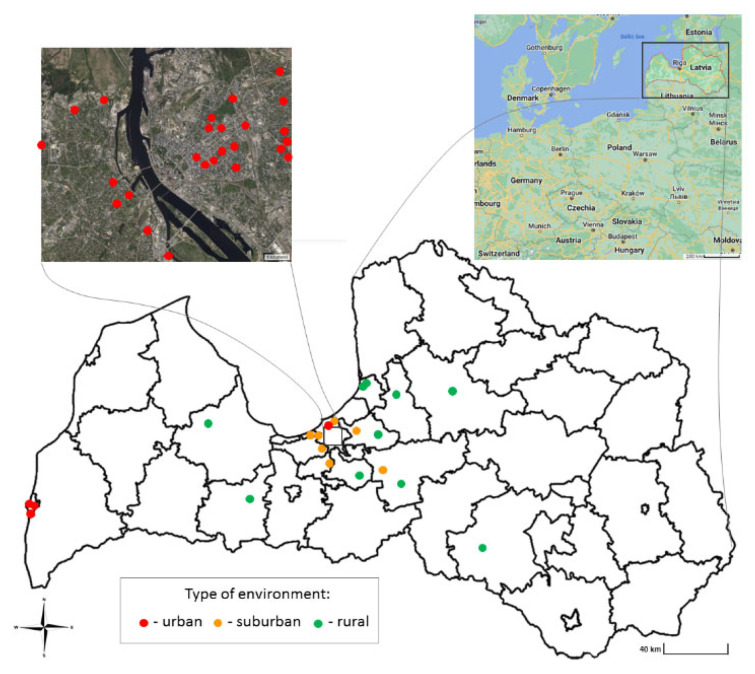
Site map of Latvia and Riga with the different types of environments represented by colored dots: red dots—urban, orange dots—suburban, and green dots—rural sampling sites [27].

The current study was conducted in Latvia (Figure 1), which is a country in the Baltic region in the northern part of Europe, covering an area of 64,589 km^2^ with a population of 1.9 million. Part of the studied residential indoor dust was collected in the capital of Latvia, Riga, which is a significantly larger city than the others with a population of 671,000 inhabitants. Riga’s territory covers 307.17 km^2^ and lies 1–10 m above sea level, on a flat and sandy plain. The average values of the air quality index (AQI) during the sampling period in the Riga central area ranged from 76 to 88 (poor air quality in terms of PM_10_, but good to very good air quality in terms of SO_2_, NO_2_ and O_3_) with a maximum of 198 [25,26]. The remainder of the dust samples were collected in four smaller Latvian cities (Liepaja, Jurmala, Ogre and Marupe) and ten rural areas (Dobele, Baldone, Kandava, Krimulda, Lielvarde, Ropazi, Saulkrasti, Zvejniekciems, Viesite and Velmeri) with a population more than 10 times less than that of the capital city.

### 2.2. Sampling Strategy

Sampling was performed between March and May 2022 at 46 sites to cover various areas in Latvia. One hundred twenty residential indoor dust samples were collected from 28 urban dwellings in a commercial area with heavy city traffic nearby, 8 suburban homes and 10 rural residences surrounded by less industrial infrastructure and traffic, but a lot more greenery. The choice of sampling sites, the number of samples per dwelling and the choice of the room where sampling was performed were based on the inhabitants’ willingness to take part in the current study and their possibilities. The participants were invited to deliver collected indoor dust samples along with the completed short questionnaire about their dwelling environment. The aggregated data of the questionnaires are summarized in Table 1, and they include parameters characterizing housing, the number of occupants, their hobbies and habits, age of the property and type of construction materials used, type of fuel used for heating and presence of pets.

Two sampling methods were applied for indoor dust collecting: household vacuum cleaners with an unused dust container for each sample, and a previously cleaned brush with a plastic spatula. Both dust sampling methods in parallel were used in 16 dwellings. In 23 dwellings, dust samples were obtained by manual sweeping, while in the remaining 7 residences, vacuuming was used for dust collection. The numbers of samples collected in each type of room from the different sampling sites are presented in Table 1, as well as in the Supplementary Materials Table S1. Residential indoor dust samples were collected from dwelling floors, carpets, windowsills, tables and shelves, and included lamp covers and other surfaces that had not been cleaned for some time. Immediately after collection, each dust sample was transferred to an unused, labeled sealed plastic bag for safe transportation and storage, avoiding cross-contamination.

### 2.3. Chemical Analysis of Indoor Dust

Prior to chemical analysis, the manual removal of pet and human hair, as well as larger refuse, was performed. Afterward, the dust samples were sieved through a 0.2 mm sieve (Rotilabo). An amount of ~0.20 g of each air-dried dust sample was subjected to conventional microwave-assisted acid digestion (2.00 mL conc. H_2_O_2_, for trace analysis, Fischer Chemical, 30% and 6.00 mL conc. HNO_3_, trace metal grade, Fisher Chemical, 69%) performed with a microwave oven (Milestone Start E) under pressure conditions. The maximum pressure at the peak point was approximately 200 psi (13.8 bar). The heating program was set as follows: heating for 15 min to 160 °C and holding at 160 °C for 30 min. Acidic extract solutions of dust samples were diluted to a volume of 15.0 mL with deionized water (0.055 µS cm^−1^, Adrona) and measured by inductively coupled plasma mass spectrometry (ICP-MS, Agilent 8900 Triple Quadrupole). The instrumental parameters of ICP-MS were set as summarized in Table 2.

For the purpose of elemental quantification, the calibration graph was constructed using six standard solutions in a concentration range from 0.1 µg L^−1^ to 100.0 µg L^−1^, which contained all the elements of interest (Al, As, Ba, Bi, B, Cd, Ca, Ce, Cr, Co, Cu, Fe, La, Pb, Rb, Li, Mg, Mn, Ni, K, Se, Na, Sr, V and Zn) dissolved in 2% HNO_3_. Analytical standard stock solutions were prepared from a certified reference material (HPS, ICP-MS-68A, 10 mg L^−1^, traceable to NIST SRM 3100). The element concentrations in the samples were calculated using the external calibration graph method, and a blank correction for each sample was applied. When dust samples were prepared using microwave-assisted acid digestion performed with a microwave oven, each set consisted of 10 samples, including one designated as a blank sample. The blank sample solely contained added reagents (H_2_O_2_ and HNO_3_), and the blank correction was applied to all samples, irrespective of the sampling method employed. The blank levels in our study for most elements (Li, V, Cr, Mn, Co, Ni, Cu, Zn, As, Rb, Sr, Cd, Ba, La, Ce, Pb and Bi) were determined to be below the limit of detection (<0.1 mg/L). However, in the case of B, Na, K, Ca and Fe, some background levels could reach up to 100 µg/L. An internal standard solution (10.0 µg L^−1^, Agilent) was used for system stability control during the measurements. Two standard solutions (10.0 µg L^−1^) were used between every ten samples to verify system stability. Two standard reference materials were used to verify the accuracy of the sample preparation and the elemental quantification methods: the standard reference material A2 (LGC, Elements on Filter Media/Work-room Air, Filter No. A2-1365) and the standard reference material B4 (LGC, Elements on Filter Media/Work-room Air, Filter No. B148).

### 2.4. Statistical Analysis

Statistical analysis was performed using Microsoft Excel version 2304 and SPSS software version 29.0. Metallic element concentration data were initially explored by descriptive statistics (arithmetic mean, median, standard deviation and coefficient of variation). Further, the Kruskal–Wallis test, which is a non-parametric statistical test, was used to determine the statistically significant differences between the medians of metallic element concentrations of the different dust sample groups. These dust sample groups were divided based on the sampling sites (urban, suburban and rural) and different premises within the dwellings. Statistically significant differences between dust samples divided based on indoor smoking habit were assessed using the Mann–Whitney U test, which is a non-parametric test used to compare two independent samples. To evaluate the statistically significant differences between concentrations of metallic elements of the dust samples collected by two sampling techniques, the Mann–Whitney U test was performed. Besides that, for the mutual comparison of the results of the samples collected using in parallel both sampling techniques, the relative differences in the determined concentrations of metallic elements were calculated. The relative differences for each of the selected 16 dwellings were obtained using the mathematical equation 1, where C_M_ is the concentration of a metallic element in a manually collected dust sample and C_V_ is the concentration of a metallic element in a sample obtained by vacuuming.

(1) $$∆D=\frac{C_{M}-C_{V}}{C_{\mathrm{mean}\left(M,V\right)}}$$

In the current study, principal component analysis (PCA), which is a well-established tool for the statistical treatment of multivariate datasets, was also performed. For the evaluation of the obtained indoor dust data, the chemometric (Chemometric Agile Tool (PCA (R-based Chemometric Agile Tool (CAT software), R version 3.1.2 (31 October 2014), University of Genoa, Italia)) approach was used with the aim to determine a collection of linear combinations of the original variables, also known as principal components, which can capture the most variation in the data. During ICP-MS analysis, data processing and collection and calculation of results were carried out using the MassHunter Workstation program, including its subprograms—Instrument control and Offline data analysis.

## 3. Results

### 3.1. Concentrations of Major and Minor Elements in Residential Dust

The metal contents of 120 residential dust samples from 46 dwellings in Latvia are summarized in Table 3. In general, the concentrations of metals in the residential dust varied significantly across the indoor locations, as determined by the coefficients of variation (CVs) and the arithmetic mean and median concentrations. Ca had the highest mean concentration, followed by Na > Mg > K > Fe > Al > Zn. From those, the most studied elements in household dust are Fe, Al and Zn, as they are more closely associated with anthropogenic sources, in contrast to Na, K, Mg and Ca, which are mostly of natural origin. For example, Ca is the major element of the mineral calcite, which is often detected in indoor dust [28]. Regarding the concentration ranges of Al and Fe, only results from Malaysia [29] reported a lower mean concentration of Al at 1230 (mg kg^−1^), compared with the current study, but data obtained from other studies were even one order higher [7,30,31]. The mean values for Zn from this study compare well with published data from Turkey [23] and Poland [32], but are several times higher than the concentrations found in Saudi Arabia [20] and Malaysia [29]. Higher concentrations of Al and Zn in Latvia could be attributed to the fact that the sampling took place in the spring when the resuspension of street dust is more pronounced. This could also be a significant reason for Al contributing to the composition of indoor dust, together with Zn from automobile emissions, i.e., the wear and tear of rubber tires and galvanized vehicular parts [8,12].

Zn is one of those elements to which attention is paid in studies on the toxic components in indoor dust, and in this study, Zn has one of the highest coefficients of variation (CVs), while the concentrations of other previously described elements were more homogeneous. In similar studies investigating exposure to toxic contaminants in indoor dust, besides Zn, which was already highlighted above, attention more often focuses on Pb, Cu, Cd, Cr, Ni, Mn and As. Among these elements, Mn and Cu had the highest mean concentration (117 mg kg^−1^), followed by Pb (61.2 mg kg^−1^), Cr (53.1 mg kg^−1^), Ni (21.5 mg kg^−1^), As (2.19 mg kg^−1^) and Cd (0.99 mg kg^−1^). Pb, Cr and Ni were detected in all collected 120 dust samples, whereas As and Cd were present in 81% and 49% of the residential dust samples, respectively. In general, compared with data obtained from around the world, these results were consistent only with reported results from Turkey (except for Ni (263 mg kg^−1^) [23]) and results from Australia (except for Pb (386 mg kg^−1^) and Cd (4.40 mg kg^−1^) [33]). It is noteworthy that concentrations of the metallic elements listed above were lower in Latvia than those reported for other regions of the world [6,13,34,35]. The arithmetic mean concentrations of other less abundant trace elements are presented in the following descending order: Ba > B > Sr > Bi > Ce > Rb > La > V > Li > Co > Se.

### 3.2. Different Sampling Approaches

It is well known that the sampling methodology is fundamental in obtaining representative samples. Variations in indoor dust surface sampling practices should be of special concern since the sampling process might reflect greatly on measurement uncertainty. If the chosen sampling method is not standardized, analytical results from different studies might not be comparable. A standardized indoor dust collection method [36,37] is not always available. In order to cover a sufficient number of indoor areas, studies have used the following indoor dust collection techniques most frequently:

- Filters from heating, ventilation and air conditioning systems [20,38];
- Dust collection from the dust bags of domestic vacuum cleaners and electrical brooms [6,8,23,31,32,33,34,39,40];
- Usage of high-volume small-surface vacuum samplers, mini-volume samplers and fine particulate dust samplers [7,12,21,41];
- Settled dust collection with wet wipes [42];
- Dust collection by gently sweeping with a brush and plastic spatula [9], soft paintbrush [5] and fingers [43].

As one of the objectives of this study was to evaluate whether the concentrations of metallic elements in indoor dust collected using two different approaches are comparable, we collected dust samples in 16 dwellings (9 urban, 2 suburban and 5 rural areas) using both methods—vacuuming and manual collection with a brush and plastic spatula—simultaneously under the same conditions. The Mann–Whitney U test was performed to compare determined concentrations of metallic elements of the samples collected by both sampling techniques. The statistical analysis did not show a statistically significant difference except for Cd (*p* < 0.05) (see Table 4). The relative differences in the determined concentrations of metallic elements in the samples collected using both sampling techniques in parallel were used for the mutual comparison of the results from each dwelling. The relative differences (ΔDs) for each of the selected 16 dwellings are summarized in Table 4.

A distinct trend was observed in the case of Na, K Ca, and Mg, since in all 16 sampling zones, higher concentrations of these elements were determined in the dust samples collected with commercial vacuum cleaners (see Supplementary Materials Table S2). Cr and Cu had the lowest relative concentration differences between the two samples, obtained in parallel using both sampling approaches. The Mn, Fe, Co, Ni and Pb concentrations were, on average, 30% higher in the samples collected by vacuuming (see Table S2). In contrast, higher concentrations of Zn were observed in the swept dust samples. In these parallel samples, Cd was the only element detected only in the dust samples collected by the vacuum cleaners. Cd is associated with fine particles [44,45,46], while Na, K and Ca are associated with larger particles in both outdoor and indoor dust [47,48,49]. Therefore, using a commercial vacuum cleaner might ensure more efficient dust sampling than collection by dust sweeping.

### 3.3. Distribution of Elements Depending on the Sampling Site and Zones within the Dwellings

**Figure 2:**
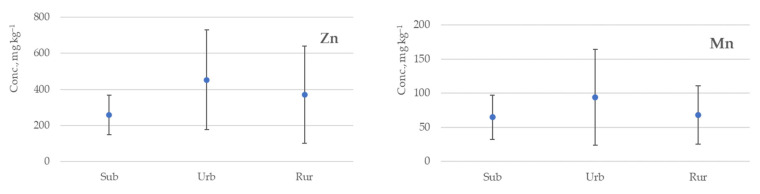

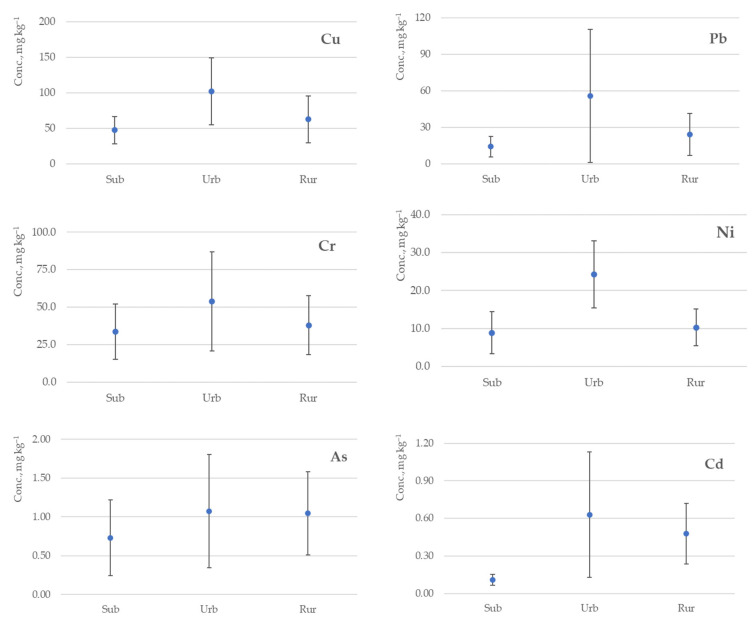
Arithmetic mean concentrations (mg kg^−1^) with the relevant standard deviations of selected metallic elements in different sampling locations: urban (Urb) (*n* = 50), suburban (Sub) (*n* = 23) and rural (Rur) (*n* = 31).

The metallic element concentrations in residential dust vary between the zones of a given dwelling and among geographical locations. Variations in the concentrations of metallic elements in different geographical locations are more representative of the impact of the outdoor sources associated with surrounding industrial sites or with increased vehicle emissions in densely populated areas. In Figure 2, arithmetic mean concentrations with the relevant standard deviations of selected metallic elements are summarized, depending on the splitting of indoor dust sampling sites into urban (*n* = 50 samples), suburban (*n* = 23 samples) and rural (*n* = 31 sample). In the calculation of arithmetic mean concentrations, the results of 16 dust samples obtained from 15 apartments whose residents smoked were not included. In addition, data were subjected to the Kruskal–Wallis test, which showed that statistically significant differences (*p* < 0.05) were observed only between the suburban–urban and suburban–rural dust sample groups for Al (*p* = 0.01 and *p* = 0.03) and Sr (*p* = 0.02 and *p* = 0.001) (see Supplementary Materials Table S3).

The concentrations of all the above-listed metallic elements, depending on the dust sampling location, have the following increasing trend from suburban sampling sites to rural and urban: Sub < Rur < Urb. Meanwhile, the content of As is quite similar in urban and rural locations. Comparatively high concentrations of As can manifest in rural sites due to metal-containing pesticides or soil fertilization. Such agricultural activities are common in the rural regions of Latvia, and this is evidenced by the fact that the target values of not only As, but also the Zn, Ni and Cd content in the soil, exceeding which cannot ensure sustainable soil quality, have been exceeded both in the topsoil and in the deeper soil layers [50]. Elevated levels of Zn and Pb in urban sites can be associated with more intense vehicular traffic and industrial activities in the cities [51]. Latif et al. found that the concentrations of Pb and Zn decreased as the distance of the houses from major roads increased in urban areas [39]. In the current study, a similar trend was observed—in dust samples from dwellings located in the center of Riga, average values of Zn (515 mg kg^−1^) and Pb (23.5 mg kg^−1^) were higher than values of Zn (390 mg kg^−1^) and Pb (5.36 mg kg^−1^) in dwellings located apart from intensive traffic streets.

**Figure 3:**
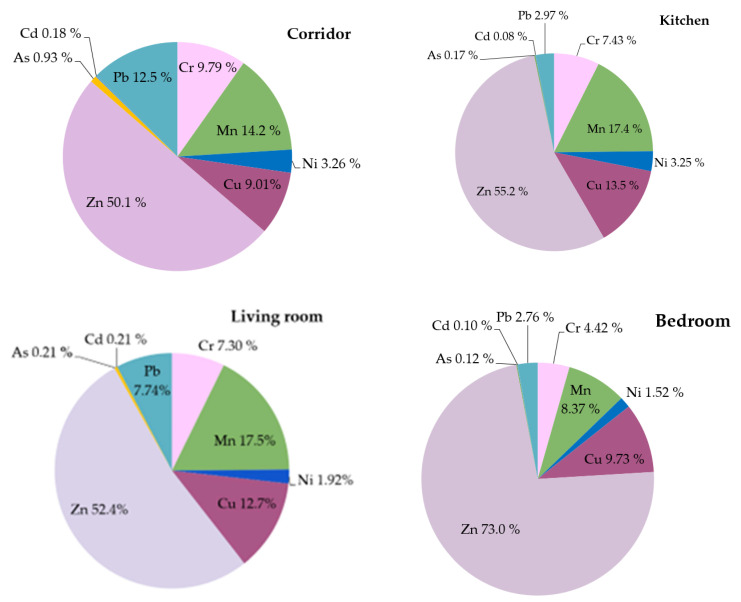
Percentages of selected metallic elements in indoor dust gathered from different rooms within dwellings (corridor *n* = 6 samples; kitchen *n* = 10 samples; living room *n* = 33 samples; bedroom *n* = 45 samples).

Dissimilarities in element distribution among different sampling rooms can indicate various internal pollution sources, which are largely affected by the household customs of the inhabitants, as well as the age of the particular building and the materials used in construction or renovation. In Figure 3, the percentages of selected metallic elements in the indoor dust of samples gathered from different rooms (corridor *n* = 6 samples; kitchen *n* = 10 samples; living room *n* = 33 samples; bedroom *n* = 45 samples) within dwellings are compared, except 5 dust samples collected from whole dwellings and 21 samples collected in the kitchen and living room together. A more detailed allocation of the metallic element concentrations obtained from different dwelling zones, in each urban, suburb and rural location, separating dwellings with smoking inside is summarized in Supplementary Materials Table S1. For all zones of dwellings, Zn accounted for the highest percentage of selected metallic elements in the bedroom area, and consequently, the percentages of the rest of the selected elements in this zone were lower. The use of various zinc-containing products, such as bed linen detergents or skin care products, which are used before bedtime, could contribute to increased concentrations of zinc. Looking closer, the mean concentrations of Zn in bedrooms located in urban, suburban and rural areas were 826 mg kg^−1^, 754 mg kg^−1^ and 701 mg kg^−1^, respectively, while in kitchens and living rooms, the concentrations of Zn were 2–3 times lower, except one urban corridor area, which had an increased value (see Table S1). The entry corridor was the area with the highest average percentages of Pb, Cr and As.

The percentages of Pb and As in the entry areas were around 3 times higher than those in the bedroom or kitchen areas, although these elements are not typically associated with predominantly outdoor origin dust, which is tracked indoors by adhering to footwear, containing elements such as Mg, Al, V, Mn and Fe [30]. The results of the Kruskal–Wallis test demonstrated statistically significant differences between dust samples from bedrooms and kitchens only for B (*p* = 0.01), Mg (0.004) and Mn (0.003). Statistically significant differences in B between samples from living rooms and kitchens (*p* = 0.01) as well as kitchens and corridors (*p* = 0.01) were also observed, probably due to the application of B-containing bleaches and detergents (see Table S4). Relatively higher concentrations of Cd were observed either in entrance corridors or in living room areas, double those concentrations found in kitchen and bedroom zones. The kitchen areas had the highest concentrations of Mn, Cu and Ni (see Table S1). The influence of cooking and smoking activities has been indicated in indoor dust with regard to the amount of particulate matter and higher amounts of Cd and Ni [39,52]. In the current study, the smoking effect was observed by comparing the average concentrations of the metallic elements determined in the dust of non-smokers’ (104 samples) and smokers’ apartments (16 samples). Elevated mean concentrations of V (7.19 mg kg^−1^), Cu (290 mg kg^−1^), Zn (1090 mg kg^−1^), As (1.39 mg kg^−1^), Cd (1.52 mg kg^−1^) and Pb (187 mg kg^−1^) in indoor dust obtained from dwellings with smoking inside were observed (see Table S1). The performed Mann–Whitney U test rejected the null hypothesis only in the case of Cr (*p* = 0.004) and Pb (0.003) (see Table S5). The literature review revealed the relationship between house age and dust heavy metal concentrations, which was significant for Cd, Pb and Zn (*p* < 0.001), but not for As, Cr, Cu or Ni [34]. Another investigation linked increased concentrations of As, Cu, Pb and Zn in indoor dust from homes > 50 years old compared with homes < 50 years old [17]. However, in the current research, the dust results from four old, segregated buildings (without smoking inside) indicated increased values. Elevated concentrations of Cr were observed in two of the buildings, with a mean concentration of 111 mg kg^−1^. High concentrations of Cu, Cd and Pb were found in three of the buildings at 116 mg kg^−1^, 2.70 mg kg^−1^ and 160 mg kg^−1^, respectively, whereas elevated concentrations of Zn (2610 mg kg^−1^) and As (5.63 mg kg^−1^) were detected in all four houses. Cd-, Pb- and Cr-containing paints or construction materials with coatings containing trace metals used in older buildings can contribute to higher levels of these elements [33,53]. Along with this, the combined effect of other mixed indoor sources (fumes from heaters, cooking, carpets or other textiles) has a significant impact [54], most notably since the presence of finer particles indoors creates an additional opportunity for contaminants to chain to dust, resulting in increased concentrations of trace elements [17].

### 3.4. Principal Component Analysis

**Figure 4:**
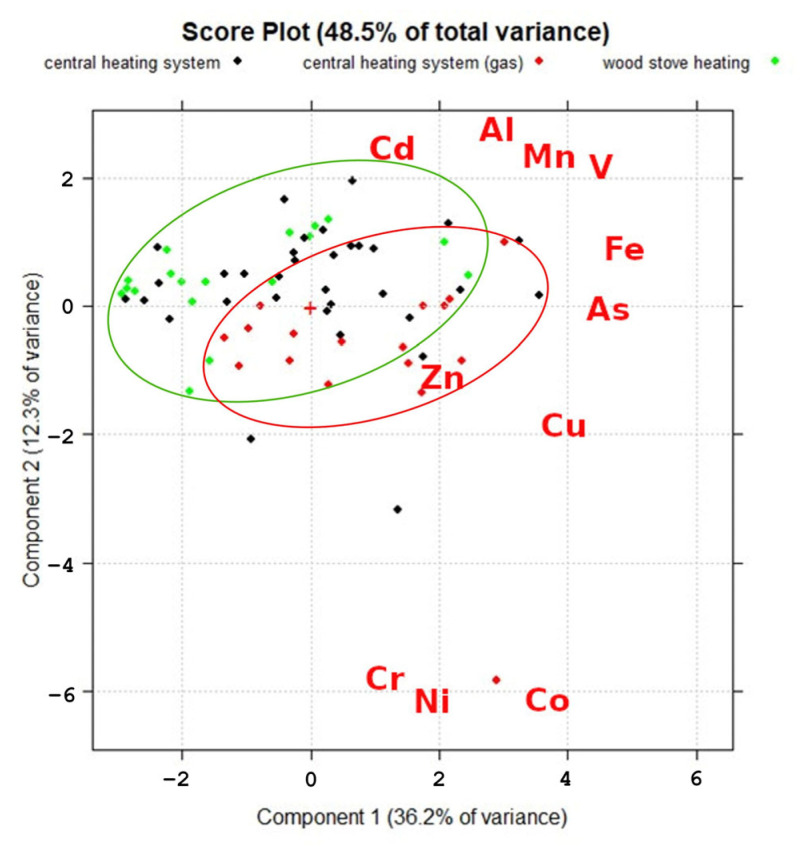
Principal component analysis of selected metallic elements in indoor dust gathered from dwellings with different heating systems.

In this study, experimental data from 120 samples were analyzed for 25 elements (Li, B, Na, Mg, Al, K, Ca, V, Cr, Mn, Fe, Co, Ni, Cu, Zn, As, Se, Rb, Sr, Cd, Ba, La, Ce, Pb and Bi) across three sampling locations with central heating systems, central heating systems with a gas stove and wood stove heating, using PCA (CAT software, manufacturer, city and country). PCA can help to decrease the dimensionality of a dataset while retaining critical information by detecting and retaining the principal components that capture the most variation in the data [55]. Concentration of chemical elements is the loading parameter for this PCA, which shows the influence extent of each element of the PCA score plot (samples). The further away from the center the element is, the more it affects the resulting score. Current results of PCA showed that the overall deviances between the samples can be characterized by component 1 (36.2%) and component 2 (12.3%). The obtained PCA plot, shown in Figure 4, shows that most differences between the groups can be observed in component 2, where all studied elements are separated into three groups: Cd, Al, Mn, V and Fe in the first group; Zn, As and Cu in the second; and Cr, Ni and Co in the third group, which had a negative correlation with the first group. In addition, PCA showed that samples from dwellings with central heating systems operated with gas tended to differ from the samples from dwellings with wood stove heating. The main differences between these two groups were in the concentrations of Zn, Cu, Cr, Ni and Co, which were lower in the dwellings with wood stove heating, where the concentrations of Al, Mn, Fe and As were higher, as explained by the higher amount of these elements in wood.

## 4. Discussion

Even though soil is considered a major contributor to household dust, its characterization is a challenge due to the complexity of the many variations and combinations of its natural and anthropogenic origin, which differ from one residential space to another [56]. In this study, higher than average concentrations were detected for the elements Ca > Na > Mg > K > Fe > Al in residential dust. These elements are major components of the Earth’s crust and natural soils, but other additional contributing sources have been observed. Based on an examination of major element correlation, Zararsiz and Öztürk reported that Al has a different origin from other crust metals [8]. For the most part, in the analyzed samples in the current study, the average range of Al concentrations varied from 1700 to 2200 mg kg^−1^, but in six dwellings, 3 to 5 times higher concentrations of Al were observed in the living rooms, bathrooms and kitchens. Additional contributors to Al sources indoors can be pharmaceuticals, cosmetics, catalysts, pigments, paints, packaging and building materials [8]. The wide use of Ca in various cosmetic products and food additives has been reported, and accordingly, Ca, along with Al, could originate from the use of cosmetic products indoors [57]. Furthermore, the average concentrations of all the previously mentioned major elements along with Mn tend to be 1.2 to 1.7 times higher in homes with pets. This could be attributed to outdoor dust tracked indoors by pets, since traditionally in Latvia it is customary to take off outdoor shoes immediately after entering the premises of dwellings.

Indoor dust can act as a medium for the deposition of metallic elements. Among other contaminants, heavy metallic elements are of concern in the context of human health issues, considering their toxicity, low degradation potential, and ability to enter and accumulate in the body [18,19]. Considering the above, in combination with the health risk evaluation using the hazard index [14], we focused on Pb followed by Zn, Cu, Cd, Cr, Ni, Mn and As as the elements for exposure research on indoor dust. The highest concentrations of these metals were observed in urban areas. Concentrations of Mn, Ni, Cu, Pb and Cr in urban zones were roughly twice as high as their concentrations in suburban and rural areas. The exceptions were As, Zn and Cd, with quite similar concentrations in urban and rural areas. Mn and As indicated the lowest concentration differences in terms of different geographical locations: 94.0 mg kg^−1^ (Urb) > 67.7 mg kg^−1^ (Rur) > 64.6 mg kg^−1^ (Sub) and 1.08 mg kg^−1^ (Urb) > 1.05 mg kg^−1^ (Rur) > 0.73 mg kg^−1^ (Sub), respectively. Zn, Cu and Mn were the elements with the highest contribution from among the above set of metallic elements, i.e., Pb, Zn, Cu, Cd, Cr, Ni, Mn and As. Contrary to studies postulating that Cu and Zn are linked to industrial and traffic sources, such as the wear and tear of tires, lubricating oils, and the corrosion of alloys and galvanized vehicle parts [8,12,58], Dingle et al. [30] reported a higher indoor contribution. This could be related to the extensive use of both Cu and Zn in household items and in construction materials [30]. In other research, Mn and Zn, along with Pb and As, were significantly associated with the concentration levels of residential soil, in contrast with Cu, Cr and Ni, indicating that indoor sources may be more dominant in the relative contributions [6]. On the other hand, comparing concentrations in different dwelling zones, located in different areas, the lowest mean concentrations were mostly observed in suburban bedrooms: Cr 33.8 ± 17.0 mg kg^−1^, Mn 57.7 ± 19.1 mg kg^−1^, Ni 8.91 ± 5.54 mg kg^−1^ and Cu 59.3 ± 32.0 mg kg^−1^. Meanwhile, the highest concentrations of Cr and Mn were detected in urban kitchens at 69.4 ± 60.7 mg kg^−1^ and 156 ± 135 mg kg^−1^, respectively. For Cu, higher concentrations were observed in rural living rooms at 159 ± 354 mg kg^−1^, (see Table S1). The effect of smoking amounted to an increase in the mean concentrations of some elements several times. Specifically, V, Cr, As, Zn and Cd increased around 2 times, but Cu and Pb increased even more, at 4 and 8 times, respectively (see Table S1).

The most challenging procedure with regard to indoor dust study is sampling to obtain representative and homogeneous data. Indoor dust sampling can be achieved in several ways, thus resulting in different units of metallic element concentrations. Besides the more often used concentration units (mg metal kg^−1^ dust), metal loadings are expressed in µg metal m^−2^ floor [42]. To avoid additional errors in the sampling procedure, measurements of the surface area to be cleaned or the duration of vacuuming were not required from the volunteers who provided us with the collected indoor dust. To compare the results of the metal loadings with the metal concentrations, the metal loading expressed in µg m^−2^ needed to be combined with the dust deposition value and the dust deposition rate [34]. During the current study, using two approaches—commercial vacuum cleaner and manual collection with a brush and plastic spatula—parallel dust samples were collected. In general, the metallic element concentrations in parallel dust samples were mutually comparable, although the concentrations (mg kg^−1^) of Na, K, Ca, Mg, Mn, Fe, Co, Ni and Pb were higher in the dust samples obtained by vacuuming. Cr and Cu showed the lowest concentration differences between parallel samples, while a higher Zn content was detected in the swept dust samples. Referring to the research done by Colt et al., household vacuum cleaners could be considered a reasonable alternative to the high-volume sampler [59]. However, after testing four types of vacuum cleaners (washable filter bagless, wet, bagged and HEPA filter-equipped robot), Vicente et al. reported that vacuuming itself is recognized as a source of indoor particle generation, and since numerous models of vacuuming devices are available on the market, further investigations are necessary [60].

Pb, Cd, As and Cr are systemic toxicants that are known to induce multiple organ damage, even at lower levels of exposure [18]. Ni has an extensive range of carcinogenic mechanisms, among them the controlled expressions of certain genes, while Mn can increase oxidative stress, thereby contributing to Mn cellular toxicity. The long-term exposure of a human body to the previously listed trace elements can progressively lead to diseases such as Parkinson’s disease, multiple sclerosis, muscular dystrophy and Alzheimer’s disease [19]. 

As there are no standard values for heavy metals in residential dust, the Regulations Regarding Quality Standards for Soil and Ground [61] were used as a reference to assess the contamination levels of heavy metals in indoor dust in Latvia. In this study, only the obtained mean concentration of Zn exceeded the critical limit value (700 mg kg^−1^), while the arithmetic mean concentrations of other heavy metallic elements were below the defined critical guidelines (As, 40 mg kg^−1^; Pb, 500 mg kg^−1^; Cr, 350 mg kg^−1^; Ni, 200 mg kg^−1^; Cu, 150 mg kg^−1^; and Cd, 8 mg kg^−1^). Taking a closer look at the results of each sampling site, only the Cd concentrations in all sites were below the critical limit value, although at two locations, the concentrations were close to the critical limit value at 5.23 mg kg^−1^ and 6.37 mg kg^−1^, respectively. Corresponding critical limit values were surpassed in the following numbers of locations: As (Sub 1); Cu (Sub 3, Rur 2, Urb 11); Pb (Rur 2, Urb 2); Zn (Sub 5, Rur 8, Urb 15); Ni (Rur 1, Urb 1); and Cr (Sub 1). This means that the concentrations of As, Cu, Pb, Zn, Ni and Cr surpassed the critical limit values in 1%, 13%, 3%, 23%, 2% and 1% of all studied dwellings, respectively. Since the comparison is between obtained concentrations and the critical limit values, when remedial measures should be taken (in the case of soil), perhaps the comparison should be made more correctly with the precautionary limit values [61], which indicate the maximum level of pollution above which it is likely to have a negative impact on human health or the environment. If the WHO environmental quality standards for soils are used for the evaluation of heavy metal contamination in residential dust, the number of excesses is more critical. The mean concentrations of As, Cu, Pb, Zn, Ni and Cr exceed the corresponding permissible limits (20 mg kg^−1^, 3 mg kg^−1^, 100 mg kg^−1^, 100 mg kg^−1^, 50 mg kg^−1^, 100 mg kg^−1^ and 300 mg kg^−1^ [56]) in 1%, 30%, 13%, 60%, 3% and 8% of dwellings in the investigated areas, respectively. Consequently, the highest risks of exposure from among the selected metallic elements could be applicable to Zn and Cu, followed by Pb and Ni.

## 5. Conclusions

The present study provided the first results of metallic element contaminations in residential indoor dust in Latvia. Indoor dust samples were collected at 46 sites to cover various areas in Latvia. A total of 120 dust samples from 28 urban dwellings, 8 suburban homes and 10 rural residences were gathered and subjected to inductively coupled plasma mass spectrometry (ICP-MS) analysis. The results of the analyzed indoor dust samples showed the following trends in terms of the major and minor metallic elements:

- High amounts (10^4^–10^3^ mg kg^−1^) for Ca > Na > Mg > K > Fe > Al > Zn;
- Moderate amounts (10^2^–10^1^ mg kg^−1^) for Ba > Cu > Mn > B > Pb > Cr > Sr > Ni > Bi;
- Lower concentrations (10^0^–10^−1^ mg kg^−1^) for Ce > Rb > La > V > Li > Co > As > Cd > Se.

For the concentrations of the selected metallic elements with regard to dust sampling location, the trend Sub < Rur < Urb was representative, except for As, which had almost similar average concentrations in the urban and rural areas.

The two applied non-standardized indoor dust sampling approaches were appropriate, easy-to-implement dust sampling techniques. However, after the comparison of metallic concentrations from parallel sampling, a well-grounded conclusion about the most effective method cannot be made, emphasizing that there is a wide variety of vacuum cleaners available on the market.

The number of heavy metal limit value exceedances indicated a higher risk for the inhabitants of urban areas, given that the combined effects of outdoor and interior sources affected the majority of the metallic elements found in household dust. The higher risks of exposure to Zn and Cu were observed not only in urban areas, but also in suburban and rural regions, with the total exceeding the critical limit values in 44 samples. The risks of exposure to Ni and Pb were observed to a lesser extent in the urban and rural sampling locations, with the critical limit values exceeded in six samples.

## Figures and Tables

**Table 1:** The characteristics of the dwellings where the indoor dust sampling was performed.

Parameter		Data from Questionnaires (Number of Dwellings * or Samples in Urban (U), Suburban (S) and Rural (R) Sites)				
Sampling site		Urban 28 *	Suburban 8 *		Rural 10 *	
Structure of the building		Bricks 19 *	Concrete 23 *		Wood 4 *	
Age of building		<5 years 3 *	6–10 years 5 *		>10 years 38 *	
Type of heating		Central heating 33 *	Wood stove heating 8 *		Briquette or pellet stove heating 5 *	
Type of room	Corridor 6 (U2; S2; R2)	Living room 33 (U16; R17)	Living room and kitchen 21 (S21)	Kitchen 10 (U6; R4)	Bedroom 45 (U24; S10; R11)	All rooms 5 (U5)
Floor cover	Hardwood 8		Laminate 42	Parquet 6	Tile 16	Other 48
Smoking	No 27 *		Yes 15 *		No information 4 *	
Pets in home	No 29 *		Yes 17 *			
Cleaning frequency	Several times per week 5 *	Twice a week 7 *	Once a week 21 *	Once per two weeks 8 *	Once per month 3 *	Less often 2 *

* Number of Dwellings.

**Table 2:** The instrumental parameters of inductively coupled plasma mass spectrometry (ICP-MS).

Parameter	Setting	Parameter	Setting
RF power (W)	1550	Extraction 1 lens (V)	−5.0
Sampling depth (mm)	8.0	Extraction 2 lens (V)	7.0
Plasma gas flow rate (L min^−1^)	15.0	Omega lens (V) 7.0	−200
Nebulizer gas flow rate (mL min^−1^)	0.90	Omega bias lens (V)	−110
Makeup gas flow rate (mL min^−1^)	0.0	Octopole bias (V)	−3.0
He cell gas flow (mL min^−1^)	5.0	Cell gas flow rate (% of full scale)	20

**Table 3:** Arithmetic mean concentrations (mg kg^−1^) and other descriptive parameters of metallic elements from indoor dust (*n* = 120 samples).

	Arithmetic Mean	Min	Max	Median	Standard Deviation	CV, %
Li	3.43	0.01	15.7	2.60	3.00	87.1
B	68.7	2.94	406	41.7	71.7	104
Na	5580	692	52,000	4270	6090	109
Mg	4250	500	15,900	3590	2690	63.2
Al	2160	8.20	10,620	1660	1930	89.0
K	4210	122	12,800	3980	2030	48.3
Ca	17,970	851	57,000	14,740	12,300	68.4
V	4.95	27.0	0.01	3.93	4.60	93.1
Cr	53.1	4.93	412	41.6	48.4	91.1
Mn	117	6.82	865	74.5	133	114
Fe	3890	187	19,380	2690	3500	90.0
Co	3.35	0.26	26.6	2.53	3.65	110
Ni	21.5	0.07	513	12.4	55.2	256
Cu	117	5.71	1880	69.3	214	182
Zn	1010	53.6	21,100	372	2430	240
As	2.19	0.01	69.2	0.78	7.33	334
Se	0.44	0.01	1.61	0.29	0.41	92.7
Rb	7.07	0.20	34.9	5.92	4.80	67.9
Sr	47.6	11.1	309	35.1	43.2	90.8
Cd	0.99	0.01	6.37	0.53	1.26	127
Ba	209	7.01	1800	109	283	136
La	4.95	0.07	23.4	3.36	4.97	101
Ce	9.25	0.33	44.6	6.18	8.99	97.1
Pb	61.2	0.27	1180	21.4	138	225
Bi	12.2	0.04	421	1.11	56.6	463

**Table 4:** The relative concentration differences (ΔDs) of some metallic elements determined in indoor dust collected using vacuuming and manual collection and the corresponding *p*-values.

*p*-Values	Zn	Mn	Cu	Pb	Cr	Ni	As	Cd
	0.66	0.10	0.41	0.31	0.58	0.22	0.12	0.002
Sampling Site	ΔD							
1-Urb	1.00	−0.37	0.31	0.39	0.67	0.06	−0.48	nd (M) *
2-Urb	1.08	0.28	0.39	0.17	−0.57	0.36	−0.62	nd (M)
3-Urb	0.32	0.22	0.28	0.57	0.63	0.08	0.31	nd (M)
4-Urb	−0.39	−0.55	−0.23	−0.97	0.14	−0.59	nd (M,V)	nd (M,V) *
5-Urb	−0.54	−1.15	−0.88	−1.62	−0.11	−0.86	nd (M)	nd (M)
6-Urb	−0.20	0.43	0.20	−0.52	0.36	0.76	−0.24	nd (M,V)
7-Urb	−0.44	−1.27	−0.06	−0.51	−0.64	−1.98	nd (M)	nd (M)
8-Urb	1.44	0.23	0.62	0.30	0.62	0.15	0.97	nd (M)
9-Urb	0.24	−0.18	0.24	−0.97	−0.27	−0.37	0.08	nd (M)
10-Sub	−1.12	−0.50	−1.54	0.56	−1.75	−1.66	nd (V) *	nd (M,V)
11-Sub	0.13	0.19	−0.15	0.38	0.27	1.10	nd (V)	nd (M,V)
12-Rur	−0.13	−0.51	−0.15	−0.85	0.09	−1.47	nd (M)	nd (M)
13-Rur	−0.44	−0.42	−0.32	−1.12	0.05	−0.81	nd (M)	nd (M)
14-Rur	0.03	−0.54	−0.22	−0.75	0.13	−0.53	nd (M)	nd (M)
15-Rur	−0.26	−0.81	−0.76	−1.00	0.44	−0.81	−1.10	nd (M)
16-Rur	1.72	−0.66	1.36	0.32	−0.27	1.23	−0.24	nd (M)

* nd (M)—not detected in manual dust collection. * nd (V)—not detected in dust samples using vacuuming. * nd (M,V)—not detected in both dust samples.

## Data Availability

Not applicable.

## Supplementary Materials

The following supporting information can be downloaded at: https://www.mdpi.com/article/10.3390/ijerph20136207/s1, Table S1: Arithmetic mean concentrations of metallic elements (mg kg^−1^) with standard deviations and minimal and maximal values detected in indoor dust samples obtained from different parts of dwellings in urban (Urb), suburban (Sub) and rural (Rur) regions of Latvia. Table S2: The determined concentrations of metallic elements in the dust samples collected using both sampling techniques—vacuuming and manual collection with a brush and plastic spatula— in parallel. Table S3: Results of the Kruskal–Wallis test and the Mann–Whitney test for different dust sample groups depending on sampling site. Table S4: Results of the Kruskal–Wallis test and the Mann–Whitney test for different dust sample groups depending on sampling location within the dwelling. Table S5: Results of the Mann–Whitney test for two dust sample groups depending on smoking inside the dwelling.
