# Supplementary material for: Distribution of Minor and Major Metallic Elements in Residential Indoor Dust: A Case Study in Latvia
Source: Int J Environ Res Public Health. 2023 Jun 22;20(13):6207. doi: 10.3390/ijerph20136207 (PMC10341758; doi:10.3390/ijerph20136207)
Supplement: Supplementary file 1 [file ijerph-20-06207-s001.zip › ijerph-2307326-supplementary.pdf]

Table S1: Arithmetic mean concentrations of metallic elements (mg kg^{-1}) with standard deviations and minimal and maximal values detected in indoor dust samples obtained from different parts of dwellings in urban (Urb), suburban (Sub) and rural (Rur) regions of Latvia.

**Sampling site**		**Conc., mg kg^{-1}**																								
		**Li**	**B**	**Na**	**Mg**	**Al**	**K**	**Ca**	**V**	**Cr**	**Mn**	**Fe**	**Co**	**Ni**	**Cu**	**Zn**	**As**	**Se**	**Rb**	**Sr**	**Cd**	**Ba**	**La**	**Ce**	**Pb**	**Bi**
Urban	Living room (n=16)	3.42±2.50 (1.21-7.23)	38.0±17.8 (13.5-58.0	5400±3240 (2730-11400)	3650±840 (2770-4530)	1660±910 (700-3640)	3870±1400 (2440-6210)	19300±9600 (11000-31700)	3.80±2.67 (1.10-9.87)	61.2±24.6 (28.2-111)	74.0±40.2 (24.5-141)	3170±1540 (1170-6150)	3.60±2.45 (0.68-8.50)	54.0±145 (1.95-513)	105±89 (41.0-287)	397±134 (234-691)	0.70±0.38 (0.33-1.60)	0.23±0.13 (0.03-0.41)	5.83±0.79 (5.05-7.67)	50.8±40.2 (19.1-129)	0.92±1.10 (0.10-2.12)	181±194 (63.0-563)	4.63±2.84 (1.12-8.65)	9.03±5.15 (3.42-16.2)	52.1±74.4 (0.7-263)	2.90±2.88 (0.21-7.36)
	Kitchen (n=6)	3.13±2.04 (1.12-8.42)	132±89.2 (15.0-258)	5590±3330 (1680-12700)	5340±4400 (2820-15900)	2680±3040 (720-10600)	4330±780 (3180-5600)	18000±11700 (1290-42700)	6.23±7.93 (0.80-27.0)	69.4±60.7 (22.0-205)	156±135 (35.5-397)	4000±3490 (979-12600)	3.07±1.50 (0.82-5.25)	20.2±11.5 (2.05-38.0)	100±51.0 (26.5-174)	385±253 (118-1020)	1.05±1.02 (0.03-3.12)	0.42±0.45 (0.05-1.40)	8.72±6.55 (5.15-24.6)	45.2±49.8 (18.3-167)	0.43±0.36 (0.13-1.08)	98.2±49.1 (51.0-195)	7.03±7.88 (1.08-23.4)	14.1±16.0 (3.87-44.6)	21.6±11.6 (7.80-43.4)	5.33±7.98 (0.22-24.1)
	Bedroom (n=24)	3.04±2.94 (0.42-9.72)	100±87.3 (12.0-233)	3390±2020 (1120-8840)	2990±1620 (1160-5780)	1110±680 (270-2880)	5520±3350 (1380-12800)	10500±8800 (1040-30600)	2.73±2.44 (0.01-8.10)	35.0±19.5 (8.89-79.0)	62.8±44.0 (6.82-143)	2420±2050 (215-7170)	2.85±3.01 (0.84-11.0)	14.9±10.8 (0.07-43.0)	97.2±58.4 (27.3-247)	826±1090 (176-4090)	1.15±0.87 (0.01-3.35)	0.50±0.25 (0.08-1.10)	5.77±1.57 (2.85-9.80)	28.8±13.6 (11.1-50.2)	0.44±0.46 (0.01-1.72)	151±140 (20.3-516)	2.13±0.76 (0.07-4.33)	4.03±2.76 (1.04-8.12)	28.8±38.8 (5.71-173)	5.11±5.15 (0.41-17.5)
	Corridor** (n=2)	1.03 15.7	18.2 58.0	1860 4550	1900 5900	730 3170	2300 418	4020 33000	0.70 11.2	29.0 120	27.2 176	865 19400	0.80 9.20	1.95 26.8	31.3 175	152 12100	0.14 11.7	0.13 0.87	4.72 6.38	17.4 65.5	0.44 1.52	80.3 412	1.30 4.35	3.20 7.85	6.06 120	0.22 5.31
Suburban	Living room and kitchen (n=21)	3.42±2.76 (0.63-5.05)	62.5±73.0 (11.7-113)	4660±630 (4210-5110)	2980±220 (2830-3140)	3492±2940 (860-9140)	4980±1660 (3810-6160)	17900±11400 (9890-57000)	6.04±3.66 (2.01-11.0)	54.4±55.5 (4.93-158)	104±82.2 (37.6-243)	3792±2843 (1090-8910)	3.35±1.85 (0.65-5.05)	13.4±12.5 (0.62-34.8)	58.0±48.5 (5.71-144)	244±138 (59.2-402)	1.30±0.82 (0.68-2.40)	0.42±0.09 (0.34-0.46)	7.22±5.54 (3.78-12.0)	51.3±4.76 (46.8-55.0)	nd	170±170 (50.4-291)	3.78±4.62 (0.91-7.60)	9.04±10.3 (1.64-16.2)	17.3±10.5 (4.3-35.0)	0.32±0.07 (0.28-0.37)
	Bedroom (n=10)	4.85±2.40 (0.85-8.10)	38.0±41.2 (9.85-97.0)	16700±23540 (3430-52000)	3630±1070 (2460-5050)	2830±1720 (800-6820)	2290±340 (1840-2680)	17600±8130 (11900-29400)	4.33±1.85 (0.80-5.95)	33.8±17.0 (12-65)	57.7±19.1 (26.0-93.8)	2754±1402 (838-4680)	2.05±0.95 (0.74-4.21)	8.95±5.65 (3.65-21.0)	59.0±31.8 (28.2-132)	754±654 (206-1950)	0.64±0.55 (0.01-1.70)	0.09±0.04 (0.09-0.2)	5.05±0.95 (3.85-7.10)	61.4±16.8 (45.5-81.2)	0.06*	62.4±22.1 (44.7-95.0)	3.3-±0.51 (2.82-3.84)	5.72±0.68 (5.10-6.51)	16.4±15.4 (4.92-58.8)	0.56±0.18 (0.37-0.72)
	Corridor** (n=2)	0.30 3.31	14.4 406	4320 4800	870 4960	450 1800	2390 4030	4220 31700	0.83 5.78	24.2 44.0	31.3 101	627 4345	0.44 4.90	1.05 10.6	35.0 58.6	84.0 196	0.92 1.40	0.10 0.12	3.94 6.05	13.8 116	0.20 1.05	32.4 91.0	2.83 3.40	5.02 7.85	0.44 41.0	2.10 3.05
Rural	Living room (n=17)	2.40±1.55 (0.33-4.60)	71.2±90.0 (14.1-230)	4260±1290 (2890-6200)	3340±1700 (870-5600)	1140±930 (330-2740)	4990±960 (4030-6080)	15300±7140 (4220-23800)	2.80±2.65 (0.10-7.12)	38.0±28.5 (16.5-92.0)	67.5±53.5 (16.0-163)	2050±1630 (187-4630)	2.01±1.40 (0.35-4.53)	9.05±5.65 (0.95-19.0)	159±354 (30.3-1100)	226±160 (84.4-495)	0.64±0.60 (0.12-1.40)	0.40±0.28 (0.01-0.70)	7.88±2.02 (3.73-10.0)	34.3±22.6 (14.0-72.4)	0.43±0.37 (0.22-1.04)	260±376 (32-920)	5.04±2.24 (2.85-7.08)	8.63±4.02 (4.92-13.0)	10.3±15.5 (0.27-39.1)	1.40±1.19 (0.16-3.09)
	Kitchen (n=4)	2.31±1.80 (0.01-5.44)	51.5±40.2 (2.94-117)	5140±4130 (690-11000)	4570±2980 (500-9440)	1850±1190 (260-3860)	3460±2034 (122-6080)	14600±10100 (2860-32500)	4.82±3.50 (0.36-11.3)	38.3±18.8 (12.0-66.2)	111±72.4 (7.95-233)	4195±3879 (327-12800)	2.60±2.05 (0.26-5.70)	17.2±23.0 (3.85-74.4)	60.2±47.1 (18.0-170)	260±173 (57.0-520)	1.20±0.60 (0.46-2.02)	0.64±0.55 (0.11-1.61)	6.06±4.13 (0.20-12.3)	33.4±8.79 (21.2-47.0)	0.53±0.27 (0.32-1.04)	180±291 (7.01-926)	3.21±2.63 (0.24-6.60)	6.80±5.11 (0.33-12.7)	17.1±15.3 (0.78-39.2)	0.96±0.70 (0.16-1.99)
	Bedroom (n=11)	2.20±1.75 (0.50-5.72)	42.5±20.3 (8.88-83.0)	4280±2330 (1520-7870)	3600±2300 (1130-7060)	1840±1340 (6810-4490)	4650±1420 (2810-7050)	16800 ±12300 (851-33900)	4.40±3.85 (0.85-12.4)	51.5±20.0 (30.7-85.2)	94.0±77.5 (28.6-255)	3150±2280 (856-7640)	3.55±1.68 (0.70-6.61)	13.3±4.55 (7.02-20.1)	85.0±38.2 (24.5-145)	701±652 (103-2524)	1.01±0.58 (0.20-2.14)	0.60±0.48 (0.13-1.50)	7.44±4.05 (2.97-14.1)	29.7±12.0 (13.5-51.3)	0.61±0.45 (0.34-1.67)	215±270 (40.0-983)	3.01±2.93 (0.34-6.70)	6.35±5.03 (1.96-14.0)	23.9±18.8 (4.50-66.1)	2.89±5.06 (0.22-17.6)
	Corridor** (n=2)	1.40 3.03	19.5 40.7	3310 6190	3830 5600	1250 2490	4700 6080	15000 18300	3.96 6.05	19.5 57.6	95.0 134	2520 4360	2.98 5.03	8.95 9.97	29.5 56.4	182 495	0.55 1.04	0.33 0.68	7.85 10.2	30.3 31.5	0.14 0.30	60.4 926	5.05 6.92	4.95 13.2	8.25 39.1	0.20 1.50
Smoking inside (n=16)		3.41±2.23 (0.33-9.12)	47.0±29.6 (7.40-113)	6880±5160 (1740-22600)	5030±2930 (1040-12300)	3130±2340 (1050-10600)	4470±1830 (1430-7220)	19000±9000 (3870-37100)	6.83±4.99 (1.03-18.0)	46.9±28.1 (9.42-106)	163±196 (23.8-865)	4560±2820 (572-11200)	3.95±3.6 (0.30-13.6)	14.5±9.6 (1.30-41.9)	290±513 (29.0-1880)	1100±1530 (53.6-5600)	1.36±1.81 (0.06-7.13)	0.38±0.34 (0.04-1.50)	8.20±3.82 (2.93-19.58)	49.5±41.0 (15.6-182)	1.51±2.06 (0.05-6.37)	262±317 (24.3-983)	6.45±4.95 (1.36-20.0)	11.8±7.97 (2.10-31.5)	187±310 (4.04-1180)	8.50±28.3 (0.13-114)
Without smoking (n=104)		3.01±3.49 (0.01-15.7)	72.6±94.4 (2.94-406)	5430±2850 (690-52000)	3430±1980 (500-15900)	2010±1800 (260-8820)	3800±1600 (122-12800)	16800±11200 (851-57000)	4.71±4.00 (0.01-27.0)	37.6±17.9 (4.93-205)	104±169 (6.82-397)	3180±2490 (187-19400)	2.31±1.55 (0.26-11.0)	20.7±53.1 (0.07-513)	75.9±64.7 (5.71-287)	391±287 (57.0-1020)	0.83±1.50 (0.01-3.35)	0.48±0.29 (0.02-1.61)	7.03±5.94 (0.2-24.6)	46.6±56.0 (11.1-167)	0.68±0.65 (0.01-2.12)	141±127 (7.01-920)	4.55±3.77 (0.07-23.4)	8.49±7.16 (0.33-44.6)	23.8±20.4 (0.27-263)	2.30±3.74 (0.16-24.1)

* Detected in one bedroom;

** Only two samples;

nd—not detected.

Table S2: The determined concentrations of metallic elements in the 16 dust samples collected using both sampling techniques—vacuuming and manual collection with a brush and plastic spatula—in parallel.

Sampling site	Sample No.	Sampling method	Conc., mg kg^{-1}														
			Na	K	Ca	Al	V	Fe	Co	Zn	Mn	Cu	Pb	Cr	Ni	As	Cd
Urban	1	Man	1130	4050	1270	1850	4.80	2990	2.40	1000	94.0	174	21.0	103	20.5	0.84	nd
		Vac	3050	6410	7880	1660	5.00	5320	2.40	336	135	127	15.0	51.0	19.4	1.37	0.17
	2	Man	1430	4110	1810	1740	4.80	3760	2.70	1200	126	247	21.0	35.0	42.7	0.71	nd
		Vac	2870	4960	11800	1510	3.80	2680	2.60	361	95.0	166	18.0	64.0	29.6	1.34	0.42
	3	Man	1680	3180	1290	912	0.20	1650	2.00	960	63.0	127	16.0	36.0	23.7	0.78	nd
		Vac	5670	5600	17800	657	1.60	1860	1.00	695	51.0	96.0	8.90	19.0	22.0	0.56	0.11
	4	Man	1860	3330	2860	348	nd	215	0.90	176	7.00	27.0	7.00	22.0	1.69	nd	nd
		Vac	3560	4580	32500	437	0.20	430	0.80	260	12.0	34.0	21.0	19.0	3.12	nd	nd
	5	Man	1120	2810	850	1170	2.00	1170	1.40	283	38.0	45.0	0.66	67.0	7.42	nd	nd
		Vac	3800	4860	30500	3030	9.90	6150	3.60	492	141	114	5.90	75.0	18.5	1.00	2.10
	6	Man	1370	3150	1040	986	1.50	2650	5.40	314	39.0	55.0	38.0	92.0	4.85	nd	nd
		Vac	3160	5470	26500	704	1.10	2280	5.00	385	25.0	45.0	65.0	64.0	2.18	0.70	nd
	7	Man	2870	2010	2890	640	nd	689	nd	713	16.0	65.0	20.0	11.0	0.07	nd	nd
		Vac	3050	3650	30700	1050	2.70	3690	2.80	1120	72.0	69.0	35.0	21.0	15.1	1.34	0.76
	8	Man	2830	1380	1040	3170	10.8	19400	9.10	12100	176	175	120	74.0	27.4	11.9	nd
		Vac	4010	3960	24300	1740	6.01	8300	6.50	1990	139	92.0	89.0	39.0	23.5	4.17	1.47
	9	Man	1120	2300	4020	2880	8.11	6480	10.5	4100	119	121	60.0	41.0	17.8	2.85	nd
		Vac	4940	3210	33000	1890	6.02	7170	11.1	3220	143	95.0	173	54.0	26.2	2.65	1.71
Suburban	10	Man	1590	2180	2510	860	3.98	2240	0.70	59.0	38.0	6.00	19.0	4.93	0.57	0.73	nd
		Vac	3280	3190	3210	9140	4.79	2230	3.10	210	64.0	44.0	11.0	74.0	6.16	nd	nd
	11	Man	2290	782	2440	1810	4.48	2560	2.70	1950	66.0	62.0	13.0	65.0	20.9	nd	nd
		Vac	3590	1620	4960	1820	2.10	1610	1.60	1710	54.0	72.0	9.05	50.0	6.06	nd	nd
Rural	12	Man	1520	2810	3080	360	0.19	335	0.70	115	17.0	33.0	0.40	19.0	1.67	nd	nd
		Vac	2910	3180	18300	550	1.25	1490	1.50	131	29.0	38.0	0.95	17.0	11.2	nd	0.17
	13	Man	2460	3660	5550	330	0.32	287	19.0	117	19.0	31.0	0.30	19.0	1.40	nd	nd
		Vac	2810	4030	12600	520	0.40	327	18.0	145	29.0	41.0	0.95	18.0	3.21	0.10	0.20
	14	Man	1570	3970	2890	1160	0.85	1180	1.90	779	40.0	79.0	19.0	42.0	6.55	nd	nd
		Vac	3510	4850	11500	1190	3.10	2710	4.30	757	69.0	98.0	42.0	37.0	11.3	0.65	0.35
	15	Man	1740	3330	3170	980	1.79	1550	2.20	420	38.0	54.0	10.0	85.0	7.00	0.23	nd
		Vac	2890	4700	15000	1510	4.07	3240	4.10	547	91.0	120	31.0	54.0	16.5	0.80	0.53
	16	Man	4320	1760	3510	2590	5.95	12800	5.70	6900	118	894	36.0	51.0	309	1.55	nd
		Vac	7510	3750	18400	3860	11.3	6930	4.80	520	23.0	170	26.0	66.0	74.0	1.97	0.46

nd—not detected;

Man—manual dust sampling;

Vac—dust sampling by vacuum cleaner.

Table S3: Results of the Kruskal–Wallis test and the Mann–Whitney test for different dust sample groups depending on sampling site.

Element	**Kruskal–Wallis H**	df	Asymp. Sig.	**Mann–Whitney U**					
				**Suburban vs. Urban**	Asymp. Sig. (2-tailed)	**Suburban vs. Rural**	Asymp. Sig. (2-tailed)	**Urban vs. Rural**	Asymp. Sig. (2-tailed)
**Li**	1.68	2	0.43						
**B**	0.78	2	0.67						
**Na**	3.08	2	0.22						
**Mg**	0.45	2	0.80						
**Al**	6.96	2	**0.03**	326	**0.01**	168	**0.03**	839	0.60
**K**	4.05	2	0.13						
**Ca**	1.20	2	0.55						
**V**	0.36	2	0.83						
**Cr**	5.83	2	0.05						
**Mn**	0.75	2	0.69						
**Fe**	0.30	2	0.86						
**Co**	3.62	2	0.16						
**Ni**	4.94	2	0.09						
**Cu**	5.32	2	0.07						
**Zn**	2.02	2	0.36						
**As**	0.02	2	0.99						
**Se**	5.03	2	0.08						
**Rb**	0.52	2	0.77						
**Sr**	7.82	2	**0.02**	79	**0.02**	16	**0.001**	596	0.75
**Cd**	3.30	2	0.19						
**Ba**	2.75	2	0.25						
**La**	0.18	2	0.91						
**Ce**	0.78	2	0.68						
**Pb**	5.32	2	0.07						
**Bi**	3.64	2	0.16						

The significance level is 0.050.

Table S4: Results of the Kruskal–Wallis test and the Mann–Whitney test for different dust sample groups depending on sampling location within the dwelling.

				**Mann–Whitney U**											
	**Kruskal–Wallis H**	**df**	**Asymp. Sig.**	**Living room vs. Bedroom**		**Living room vs. Kitchen**		**Living room vs. Corridor**		**Bedroom vs. Kitchen**		**Bedroom vs. Corridor**		**Kitchen vs. Corridor**	
**Li**	3.51	3	0.32												
**B**	11.82	3	**0.01**	338	0.67	64.0	**0.01**	20.0	0.28	104	**0.007**	22.0	0.12	0.000	**0.01**
**Na**	7.51	3	0.06												
**Mg**	9.27	3	**0.03**	280	0.15	102	0.16	23.0	0.40	98.0	**0.004**	23.0	0.13	17.0	0.74
**Al**	3.97	3	0.26												
**K**	4.17	3	0.24												
**Ca**	3.21	3	0.36												
**V**	7.09	3	0.07												
**Cr**	2.51	3	0.47												
**Mn**	8.47	3	**0.04**	634	0.25	201	0.08	78.0	0.77	192	**0.003**	97.0	0.67	24.0	0.15
**Fe**	5.28	3	0.15												
**Co**	0.36	3	0.95												
**Ni**	5.18	3	0.15												
**Cu**	3.20	3	0.36												
**Zn**	6.17	3	0.10												
**As**	3.98	3	0.26												
**Se**	2.55	3	0.47												
**Rb**	2.49	3	0.48												
**Sr**	2.96	3	0.40												
**Cd**	0.49	3	0.92												
**Ba**	0.91	3	0.82												
**La**	7.06	3	0.07												
**Ce**	6.96	3	0.07												
**Pb**	0.53	3	0.91												
**Bi**	3.00	3	0.39												

The significance level is 0.050.

Table S5: Results of the Mann–Whitney test for two dust sample groups depending on smoking inside the dwelling.

Li	B	Na	Mg	Al	K	Ca	V	Cr	Mn	Fe	Co	Ni	Cu	Zn	As	Se	Rb	Sr	Cd	Ba	La	Ce	Pb	Bi
0.49	0.56	0.41	0.34	0.43	0.75	0.87	0.05	**0.004**	0.10	0.08	0.68	0.38	0.18	0.65	0.54	0.83	0.87	0.96	0.43	0.93	0.47	0.43	**0.003**	0.34

The significance level is 0.050.
